# An Automated Algorithm for Obstructive Sleep Apnea Detection Using a Wireless Abdomen-Worn Sensor

**DOI:** 10.3390/s25082412

**Published:** 2025-04-10

**Authors:** Thi Hang Dang, Seong-mun Kim, Min-seong Choi, Sung-nam Hwan, Hyung-ki Min, Franklin Bien

**Affiliations:** 1 Department of Electrical Engineering, Ulsan National Institute of Science and Technology, 50, UNIST-gil, Ulsan 44919, Republic of Korea; thihangdang@unist.ac.kr (T.H.D.);; 2 SB Solutions Inc., Ulsan National Institute of Science and Technology, Ulsan 44919, Republic of Korea; mschoi@unist.ac.kr (M.-s.C.);

**Keywords:** obstructive sleep apnea, MLP-mixer, home sleep apnea test, abdomen-worn sensor, capacitive sensor

## Abstract

Obstructive sleep apnea (OSA) is common among older populations and individuals with cardiovascular diseases. OSA diagnosis is primarily conducted using polysomnography or recommended home sleep apnea test (HSAT) devices. Wireless wearable devices have emerged as promising tools for OSA screening and follow-up. This study introduces a novel automated algorithm for detecting OSA using abdominal movement signals and acceleration data collected by a wireless abdomen-worn sensor (Soomirang). Thirty-seven subjects underwent overnight monitoring using an HSAT device and the Soomirang system simultaneously. Normal and apnea events were classified using an MLP-Mixer deep learning model based on Soomirang data, which was also used to estimate total sleep time (ST). Pearson correlation and Bland–Altman analyses were conducted to evaluate the agreement of ST and the apnea–hypopnea index (AHI) calculated by the HSAT device and Soomirang. ST demonstrated a correlation of 0.9 with an average time difference of 7.5 min, while AHI showed a correlation of 0.95 with an average AHI difference of 3. The accuracy, sensitivity, and specificity of the Soomirang for detecting OSA were 97.14%, 100%, and 95.45% at AHI ≥ 15, respectively. The proposed algorithm, utilizing data from a wireless abdomen-worn device exhibited excellent performance in detecting moderate to severe OSA. The findings underscored the potential of a simple device as an accessible and effective tool for OSA screening and follow-up.

## 1. Introduction

Obstructive sleep apnea (OSA) is characterized by recurrent episodes of partial or complete upper airway collapse, each lasting at least 10 s. These events are typically associated with either cortical arousal or a decrease in blood oxygen saturation [1,2]. The severity of OSA is assessed using the apnea–hypopnea index (AHI), which classifies the condition as mild (5 ≤ AHI < 15), moderate (15 ≤ AHI < 30), or severe (AHI ≥ 30) [1,2]. OSA increases the risk of cardiovascular diseases [3] and affects approximately 20–60% of adults, with prevalence rising to 75% in individuals aged 65 years or older [4,5]. These statistics highlight the critical need for effective diagnosis and management of OSA.

The standard diagnostic method for OSA is polysomnography (PSG), which remains the gold standard but is costly, inconvenient, and limited in accessibility due to the requirement for specialized sleep laboratories and extended waiting times [1,2]. As a result, up to 80% of OSA cases remain undiagnosed [6]. To address these limitations, home sleep apnea testing (HSAT) has gained widespread adoption as a cost-effective and accessible alternative. The American Academy of Sleep Medicine (AASM) strongly recommends HSAT with a technically adequate device for diagnosing OSA in uncomplicated adults at moderate to severe risk [1]. In recent years, artificial intelligence (AI)-enabled wireless wearable devices, such as smartwatches, rings, and chest-worn sensors, have gained prominence as cost-effective and non-invasive tools for the pre-diagnosis and diagnosis of OSA [7]. The performance of these devices varies, with reported accuracy ranging from 70% to 100%, sensitivity from 76% to 100%, and specificity from 29% to 99% [7]. Devices worn on the chest or abdomen tend to perform better in OSA detection compared with those worn on the wrist or finger, primarily due to their ability to directly measure respiratory signals and body positions, which are key factors for accurate OSA detection. Capacitive sensors are a promising solution for wireless monitoring breathing and respiration due to their simplicity and passive operation, which does not require an external power source [8]. Accelerometers are cost-effective and efficient for monitoring motion and position, including detecting ribcage movement associated with respiration [9,10]. Previous studies have highlighted the utility of chest-worn accelerometers for awake/sleep detection, with chest and abdomen-worn accelerometers showing better performance for awake/sleep detection compared with wrist accelerometers alone [11]. A combination of a capacitive sensor and an accelerometer attached to the abdomen could be a good solution for OSA detection. In this study, an AI-enabled wireless abdomen-worn device for OSA detection was developed.

The paper is structured into six sections. Section 2 presents related works and the main contribution of this study. Section 3 describes the data collection and proposed algorithms. Section 4 shows the results. Section 5 provides a discussion and limitations of the study. Finally, Section 6 includes a conclusion and further work.

## 2. Related Works

Numerous studies have developed wearable HSAT devices for OSA screening. Various types of wired and wireless sensors have been designed for respiratory monitoring, including oximeters [12], nasal cannulas [12], respiratory inductance plethysmography [13], photoplethysmography (PPG) [14], face patches [15], capacitive sensors [16,17], and accelerometers [18]. These devices integrate AI-driven algorithms to analyze physiological signals such as airflow [12], oxygen saturation [11], respiratory effort [11,13], ballistocardiography [19], PPG [14], heart rate [14], and body movement [18], enabling efficient and accessible OSA screening and follow-up.

Wearable devices for OSA detection can be placed at different body locations, including the chest [20], abdomen [11], wrist [21,22], and finger [14]. OSA classification typically follows two approaches: apnea–hypopnea index (AHI) estimation and respiratory event-level detection. In AHI-based classification, the system estimates the AHI for an entire night’s sleep, a method frequently used in wireless wearable devices. In event-level detection, apnea episodes are identified, and the AHI is computed by dividing the number of detected apnea events by the total sleep time (ST). The ST can be estimated using an accelerometer [11,14].

Various machine learning (ML) and deep learning (DL) techniques have been proposed for OSA detection. Convolutional neural networks (CNN) [12,21], long short-term memory (LSTM) networks [23], and random forests [12] are among the most widely used approaches [7]. Deep learning models have consistently outperformed traditional machine learning approaches in this domain [7,12]. Recently, the multiscale MLP-Mixer technique demonstrated superior performance over CNN and LSTM models in computer vision tasks and OSA detection using large datasets [24,25,26]. Unlike CNN, which requires extensive hyperparameter tuning, the MLP-Mixer is lightweight, efficient, and capable of operating on devices without a graphics processing unit (GPU), while still delivering competitive performance [27]. Despite its advantages, the MLP-Mixer has been rarely explored for OSA detection. Table 1 summarizes related works and their performance in OSA detection. 

This study presents several key contributions:

1. Novel wireless sensor application: This is the first study to apply a single-point contact-based capacitive sensor and accelerometer (Soomirang) for portable OSA screening, tested through a full-night home sleep experiment with institutional review board (IRB) approval.
2. Automated OSA detection algorithm: We propose a lightweight MLP-Mixer-based deep learning model for OSA detection using data collected from Soomirang. This modified model is optimized for time-series data and was compared against the non-light weight bidirectional LSTM (BiLSTM) model in the performance evaluation. The algorithm can estimate AHI, detect respiratory events, and estimate total sleep time.

This compact, wireless wearable device integrates a capacitive sensor and a three-axis accelerometer, designed for single-point attachment to the abdomen. The sensor wirelessly captures high-quality physiological sleep signals, including respiration and body movement, which are critical for OSA detection. Additionally, a deep learning-based MLP-Mixer algorithm was developed for apnea event detection. The ST was estimated using accelerometer signals. The final AHI score was computed as the ratio of detected apnea events to ST in hours. A full-night HSAT was conducted to validate the algorithm’s performance. The results indicate that this small, wireless wearable device, enhanced with deep learning algorithms, holds significant potential for OSA screening in home healthcare settings.

## 3. Materials and Methods

**Figure 1:**
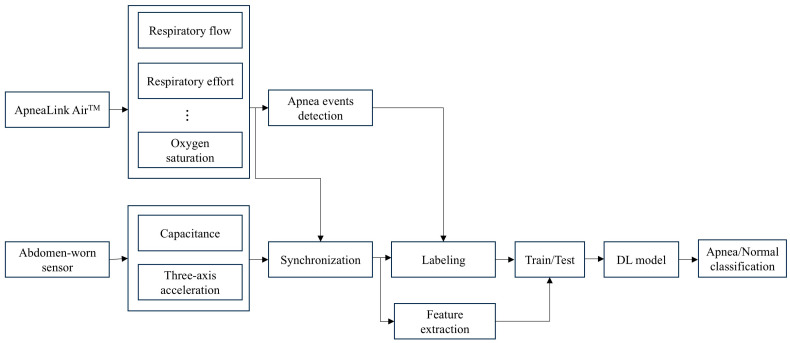
Flow chart diagram for OSA detection using Soomirang data. DL model, deep learning model.

Figure 1 shows a flow chart diagram for OSA detection and validation using the Soomirang device (SB Solutions Inc., Ulsan, Republic of Korea).

### 3.1. Data Collection

**Figure 2:**
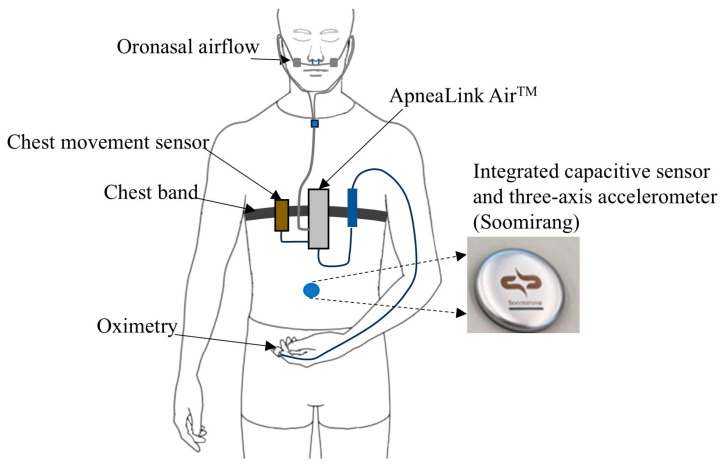
Experimental setup for overnight sleep monitoring with the ApneaLink Air^TM^ and Soomirang devices.

Thirty-seven participants were enrolled for the experiment, and written informed consent was obtained from all subjects. The study received ethical approval from the Institutional Review Board of Ulsan National Institute of Science and Technology (UNIST) under the reference number UNISTIRB-24-006-A. Each subject underwent an overnight measurement with simultaneous sleep monitoring using the ApneaLink Air^TM^ device (Resmed, Bella Vista, NSW, Australia) and the Soomirang device (Figure 2). The ApneaLink Air^TM^ device measured oronasal airflow (sample frequency: 100 Hz), thoracic respiratory effort (10 Hz), pulse, and peripheral oxygen saturation (1 Hz). The Soomirang device (SB Solutions Inc., Ulsan, Republic of Korea) was attached to the belly between the navel and the chest to measure signals of abdominal movement and three-axis acceleration at a sampling rate of 5 Hz. The abdominal movement signal was captured as changes in capacitance, reflecting variations in the distance between the device and the abdominal surface during breathing.

### 3.2. Preprocessing

The ApneaLink Air^TM^ and Soomirang data were synchronized for analysis. Breathing-disordered events were scored using the ApneaLink application (version 10.10), where each sample was labeled as either apnea (obstructive, central, mixed apnea, or hypopnea) or normal (non-apnea). The start and end times of apnea events were extracted from the ApneaLink software. Correspondingly, each Soomirang data sample was labeled as apnea if its measured time fell within the start and end times of an apnea event; otherwise, it was labeled as normal. The capacitance signal was smoothed using a Savitzky–Golay filter with a window length of 11 samples and a filter order of 3, followed by normalization [28]. The third order was selected to ensure that the filter could capture both a peak and a valley in the signal. The window length was calculated on the basis of the equation proposed by Ross et al., assuming an average respiratory rate of 15 breaths per minute (with respiratory rates during apnea episodes ranging from 0 to 30 breaths per minute) [29]. The capacitance signal within each moving window was normalized by subtracting the mean and dividing by the standard deviation of the values in the window. To capture local variations in the capacitance signal per second, the local gap was calculated as the difference between the maximum and minimum values within a five-sample moving window. Sleep position feature was extracted, and each data sample was classified into upright, supine, prone, left, and right positions using gravitational acceleration and tilt angles from a three-axis accelerometer [10]. The roll angle (horizontal rotation) was computed as the arctangent of the ratio of *z*-axis to *y*-axis acceleration, while the pitch angle (forward/backward tilt) was calculated as the arctangent of the ratio between the square root of the sum of the squared *y*-axis and *z*-axis accelerations to the negative *x*-axis acceleration. Position classification was based on specific thresholds: upright (absolute *x*-axis ≈ 1 or pitch > 109°), supine (*z*-axis ≈ −1 or pitch < 109° with roll 247.5–292.5°), prone (*z*-axis ≈ 1 or pitch < 109° with roll 67.5–112.5°), left (*y*-axis ≈ −1 or pitch < 109° with roll 112.5–247.5°), and right (*y*-axis ≈ 1 or pitch < 109° with roll −67.5–67.5°). These thresholds were empirically determined.

**Figure 3:**
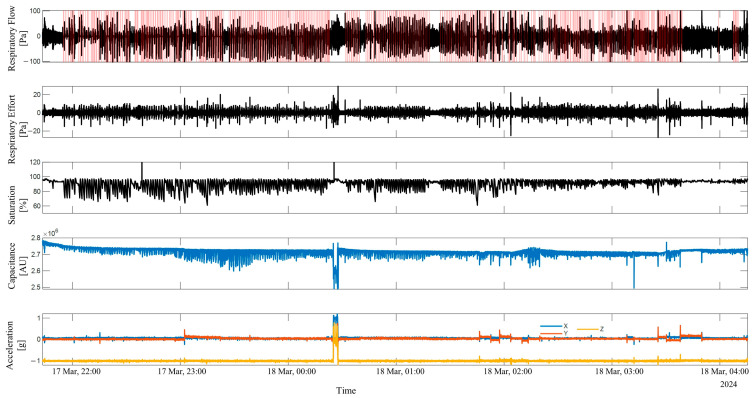
Data recordings from a full-night study of a subject were captured using the ApneaLink Air™ (respiratory flow, respiratory effort, and oxygen saturation) and the Soomirang device (capacitance and three-axis acceleration). The red areas alongside the respiratory flow signal were denoted as annotated apnea events extracted from the ApneaLink Air™.

**Figure 4:**
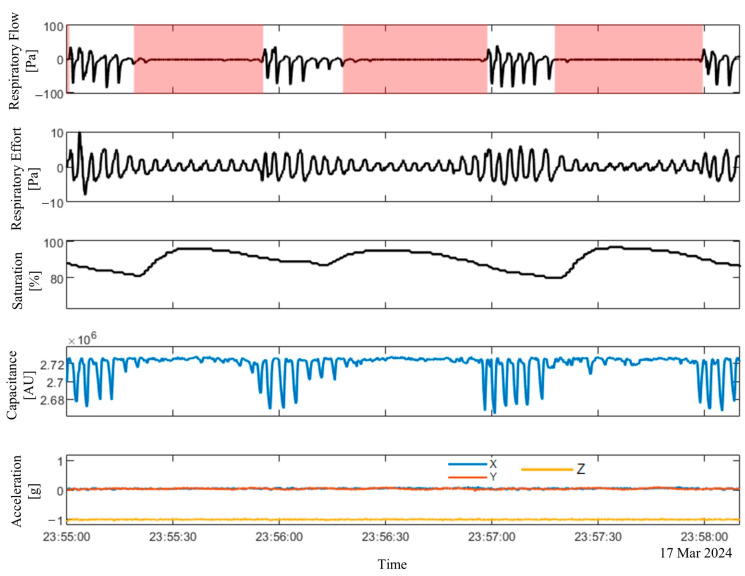
Data recordings from the ApneaLink Air™ (respiratory flow, respiratory effort, and oxygen saturation) and the Soomirang device (capacitance and three-axis acceleration) within a 100-s recording window. The red-highlighted areas along the respiratory flow signal indicate annotated apnea events identified by the ApneaLink Air™.

Additionally, the length of the acceleration vector was calculated as the square root of the sum of the squared readings from each axis. Figure 3 shows the data recording and labeling from the ApneaLink Air^TM^ (respiratory flow, respiratory effort, and saturation signals) and the Soomirang device (capacitance and three-axis acceleration) from one subject. The annotated apnea events extracted from the ApneaLink Air™ are highlighted in red areas alongside the respiratory flow signal. Figure 4 presents the data recording of a 100-s time window.

### 3.3. Models

#### 3.3.1. MLP-Mixer Model

**Figure 5:**
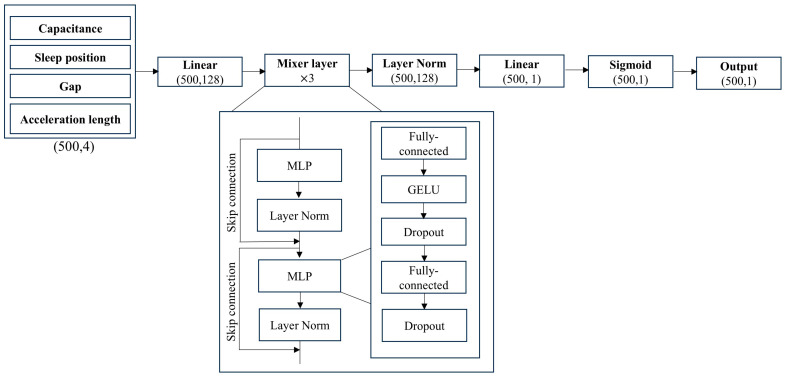
MLP-Mixer model for apnea/normal classification. GELU, Gaussian Error Linear Unit; MLP, multilayer perceptron.

The MLP-Mixer model was introduced by Tolstikin et al. for the image classification task. In this study, the MLP-Mixer model was adapted for the time series data task using the PyTorch (version 2.5.1) library [24]. The MLP-Mixer model takes input from a 100-s time window and processes the data by sliding this window across the entire sleep period, from the start to the end. As a result, the input to the MLP-Mixer model consists of 500 × 4 tables (500 patches, 4 channels). The four channels are four features extracted from the Soomirang data as described in preprocessing, which include normalized capacitance, length of the acceleration, gap, and sleep position. Each table is then transposed, and each row of the table is fed into the same MLP (token mixing). An MLP layer consists of two dense layers, two dropout layers and a Gaussian Error Linear Unit (GELU) nonlinearity [30]. All weights are shared across the same channel for different patches. Subsequently, the table is reversed and transformed back into patches, followed by the same computation being applied to all patches (second MLP layer or channel mixing). Token mixing is implemented using Conv1d with a kernel size of 1, while channel mixing is performed using a linear fully connected layer. Skip connections are used in both the token mixing and channel mixing blocks to prevent gradient vanishing phenomena during training. Sigmoid activation was added before the classification task. The model was trained using a five-fold cross-validation approach with a batch size of 500, a window size of 500, a hidden dimension of 128, and a training iteration of 50,000. The training was performed using the Adam optimizer with a learning rate of 0.001. Details of the hyperparameter tuning process are provided in Appendix A (Table A1). Figure 5 shows the MLP-Mixer model utilized to classify apnea/normal events for time series data. The sigmoid activation function was employed for each layer, and the binary cross-entropy (BCE) loss function was used to calculate the cross-entropy loss. This study aimed to compare the performance of a lightweight model (MLP-Mixer) and a non-lightweight model (BiLSTM). Therefore, both models were configured with a large batch size (500 samples) and a substantial model size for the classification task. The same number of layers, hidden units, optimizer, and loss function were kept for both models to maintain consistency in the evaluation. Further increases in batch size were restricted by GPU resource limitations.

#### 3.3.2. BiLSTM Model

**Figure 6:**
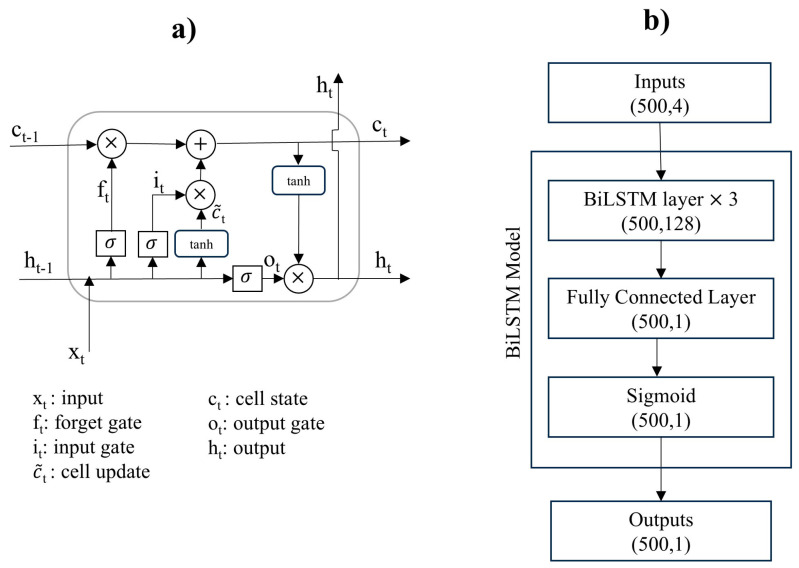
BiLSTM model for apnea/normal classification: (**a**) an LSTM cell; (**b**) BiLSTM model.

BiLSTM layers capture bidirectional long-term dependencies within the time series or sequence data. This approach is suitable for our dataset, where apnea and normal events are characterized by features of time-varying capacitance, length of acceleration, and sleep positions. BiLSTM was observed to have better prediction performance compared with the LSTM model [23,31]. Each BiLSTM layer is composed of two stacked LSTM layers. An individual LSTM cell within these layers consists of three primary gates, namely the input gate, output gate, and forget gate. These gates are optimized during training to regulate the input, output, and memory of cells. The LSTM preserves the output while combining with the new input, effectively maintaining context and handling sequential data dependencies (Figure 6a). Figure 6b shows the BiLSTM architecture used for apnea/normal event detection, comprising three main layers: the input layer, the BiLSTM model, and the output layer. The input layer contains four neurons, corresponding to the four extracted features derived from Soomirang. The BiLSTM model comprises three BiLSTM layers followed by a fully connected linear layer. To generate class probabilities, the network’s output is normalized using the sigmoid function. The model was trained using a five-fold cross-validation approach with a batch size of 500, a window size of 500, and a hidden dimension of 128. The training was performed using the Adam optimizer with a learning rate of 0.001. The sigmoid activation function was employed in each BiLSTM layer, and the BCE loss function was used to calculate the cross-entropy loss.

### 3.4. Postprocessing

#### 3.4.1. Apnea Prediction

The model outputs “apnea probability” values (*p*) for the entire sleep interval. The sensitivity of the model is determined by two thresholds: the apnea probability threshold ($\theta_{p}$) and the apnea event time threshold (τ). A data sample is classified as apnea if its *p* value is at least equal to $\theta_{p}$; otherwise, it is classified as normal. Lower $\theta_{p}$ values increase the model’s sensitivity, while higher values reduce it. The optimal $\theta_{p}$ value was determined using the Youden index from the receiver operating characteristic curve. This index identifies the point where the maximum difference between the true positive rate (TPR) and the false positive rate (FPR) is achieved [32]. The TPR represents the proportion of correctly identified apnea samples, while the FPR indicates the proportion of normal samples incorrectly classified as apnea. The area under the curve (AUC) was computed to compare the apnea detection performance of the MLP-Mixer and BiLSTM models [33]. The model with the higher AUC was selected for further steps of sleep apnea detection.

Initial apnea prediction can also include very short apnea intervals. Therefore, postprocessing is required to remove such short apnea intervals. During this step, apnea intervals shorter than *τ* are excluded. For example, given the sensor’s frame rate of approximately 5 Hz, setting *τ* to 50 removes apnea intervals shorter than 10 s.

#### 3.4.2. Sleep Time Estimation

Several studies have demonstrated a direct relationship between sleep stages and bio-signal patterns [7,14,21]. Wakefulness and sleep states can be quantified by analyzing body movements, which decrease in size and frequency as sleep progresses from wakefulness to deeper stages. A time segment was classified as being in the awake stage if it met one of the following conditions: (1) it occurred before sleep onset, (2) it occurred during the morning wake-up period, (3) it occurred during periods of an upright position. The length of the acceleration was used to calculate both sleep latency and morning wake-up period. A period without body movements was detected if the length of accelerometer values within that segment fell within a pre-defined lower and upper threshold. Sleep onset was defined as the start of the first segment with no body movements lasting for at least 10 min. The sleep latency was calculated as the difference time between sleep onset and the start of the recording time. The same approach was used to calculate the morning wake-up period, using the reversed length of acceleration. The morning wake-up period was defined as the first wake-up period relative to the end of the recording. The ST estimation was calculated as the time difference between the recording time and the awake time. The AHI estimated by the Soomirang device (AHI_Soomirang_) was calculated as the ratio between the number of detected apnea events and ST.

### 3.5. Statistical Analysis

Statistical analyses were performed using MATLAB (version 2023b, MathWorks, MA, USA). Pearson correlations and Bland–Altman analysis were employed to evaluate the agreement in the evaluation time (or ST) and AHI calculated from the ApneaLink Air™ (AHI_ApneaLink™_) and the Soomirang device. The accuracy, sensitivity, and specificity of the Soomirang device for detecting OSA were assessed in comparison with the ApneaLink Air™ at AHI thresholds of 5, 15, and 30 events/h. The formulas for these metrics were derived from reference studies [34].

## 4. Results

Thirty-seven recordings were collected for this study. After excluding two recordings due to poor data quality from the Soomirang device (one with missing accelerometer data and the other having time sampling issues), twenty-five recordings were used for training, and ten recordings were reserved for testing. According to the results extracted from ApneaLink Air^TM^, the number of subjects with normal, mild, moderate, and severe OSA were 14, 8, 3, and 10, respectively. The average AHI was 23.2 ± 28.54. The average recording time was 342.97 ± 80.95 min.

The AUC values for the MLP-Mixer and BiLSTM models were 84.28 and 81.28, respectively. The MLP-Mixer model was selected for further analysis due to its superior AUC performance compared with the BiLSTM. At the selected optimal threshold, the MLP-Mixer model achieved a true positive rate and a false positive rate of 76% and 75.95%, respectively, for apnea/normal classification.

**Figure 7:**
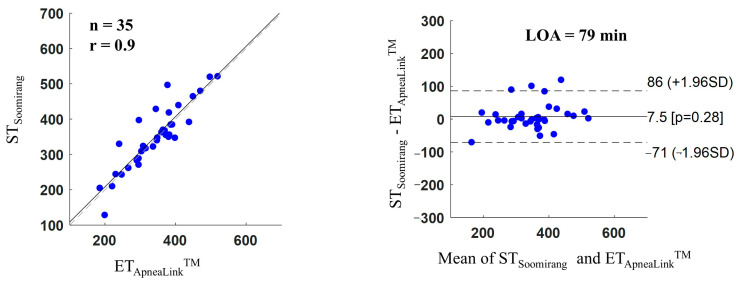
Pearson correlations and Bland–Altman plots for a comparison between estimated sleep time extracted from Soomirang (ST_Soomirang_) and evaluation time extracted from ApneaLink Air^TM^ (ET_ApneaLink_^TM^), in which n is the number of subjects, r is the correlation coefficient, LOA is the limit of agreement, and SD is the standard deviation. The dotted line in the Pearson correlation plot represents the line of equality, the dotted lines in the Bland-Altman plot indicate the range within which 95% of the differences are expected to fall.

Figure 7 shows the Pearson correlations and Bland–Alterman plots in a comparison between the estimated ST extracted from Soomirang (ST_Soomirang_) and the evaluation time extracted from ApneaLink Air^TM^ (ET_ApneaLink_^TM^). The correlation coefficient (r) was 0.9 and the limit of agreement (LOA) at 95% confidence (±1.96 SD) ranged from −71 to 86 min (mean difference of 79 min). The average time difference between the two devices was 7.5 min.

**Figure 8:**
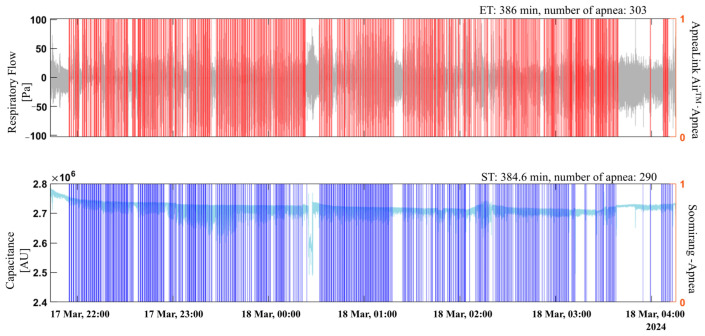
Example of apnea events detected by the Soomirang device compared with those detected by ApneaLink Air™ in an entire recording. Red and blue bars represent apnea events detected by ApneaLink Air^TM^ and Soomirang, respectively.

**Figure 9:**
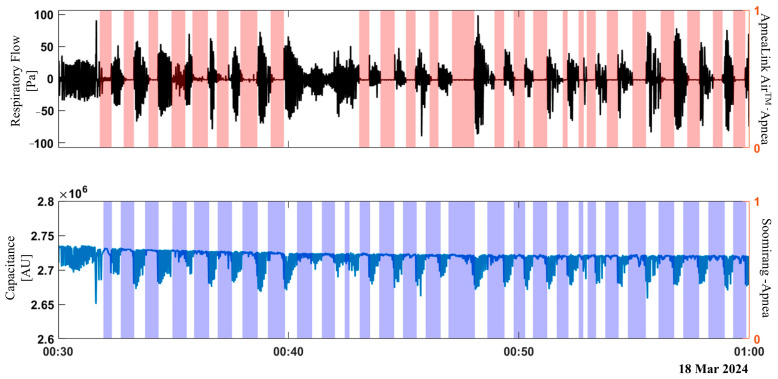
Example of apnea events detected by the Soomirang device compared with those detected by ApneaLink Air™ in a detailed view of a 30-min data window. Red and blue areas represent apnea events detected by ApneaLink Air^TM^ and Soomirang, respectively.

Figure 8 shows an example of apnea event prediction using the MLP-Mixer model for one recording from the Soomirang device compared with the apnea events detected by ApneaLink Air^TM^. Apnea events detected by ApneaLink Air^TM^ and Soomirang are represented by red and blue bars, respectively. The number of apnea events, ET, and AHI calculated by ApneaLink Air^TM^ were 303, 386, and 47.3, respectively. In comparison, the number of apnea events, ST, and the AHI calculated by Soomirang were 290, 384.6, and 45.2, respectively. The differences in the number of apnea events, ST (ET), and AHI between the two devices were 13, 1.4 and 2.1, respectively. Figure 9 provides a closer view, showing a 30-min data window.

**Figure 10:**
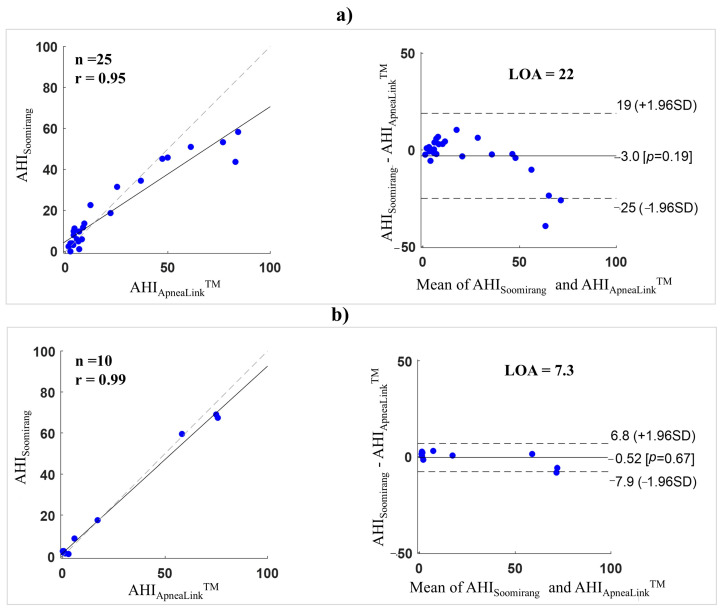
Pearson correlation and Bland–Altman plots for a comparison between the AHI calculated by Soomirang and ApneaLink Air^TM^ devices: (**a**) in a five-fold validation and (**b**) in the test set. The dotted line in the Pearson correlation plot represents the line of equality, the dotted lines in the Bland-Altman plot indicate the range within which 95% of the differences are expected to fall. The character n is the number of subjects, r is the correlation coefficient, LOA is the limit of agreement, and SD is the standard deviation.

Figure 10 shows the Pearson correlations and Bland–Alterman plots for comparison between AHI_ApneaLink™_ and AHI_Soomirang_ in five-fold validation (Figure 10a) and test set (Figure 10b). The r in the five-fold validation and test set were 0.95 and 0.99, respectively. The LOA at 95% confidence (±1.96 SD) ranged from −25 to 19 in the five-fold validation (mean difference of 22) and from −7.9 to 6.8 in the test set (mean difference of 7.3). The average AHI difference between the two devices in the five-fold validation and test set was 3 and 0.52, respectively.

The accuracy, sensitivity, and specificity of the Soomirang device for detecting OSA were assessed in a total of thirty-five patients in a five-fold validation and test set. At the threshold of AHI = 5, the accuracy, sensitivity, and specificity were 85.71%, 90.91%, and 76.92%, respectively. At the threshold of AHI = 15, the accuracy, sensitivity, and specificity were 97.14%, 100%, and 95.45%, respectively. At the threshold of AHI = 30, the accuracy, sensitivity, and specificity were 97.14%, 100%, and 96%, respectively.

## 5. Discussion

This study presents an automated algorithm for detecting OSA using data collected by a wireless abdomen-worn sensor (Soomirang), which integrates a capacitive sensor for respiratory-like data measurement and a three-axis accelerometer. This study was performed in a home setting. The study cohort was selected randomly, not from an in-lab study population, which usually has a higher risk than the general population. The main findings were (1) the new compact Soomirang device with an integrated novel automatic algorithm demonstrated excellent performance for OSA detection, especially in moderate to severe OSA patients; (2) a strong correlation was found between the ST calculated by Soomirang and HSAT (ApneaLink Air^TM^); and (3) it revealed the good performance of the MLP-Mixer model in an application for OSA detection.

Given that the Soomirang device was designed for home-based testing, the performance of this device was first compared with HSAT, which best replicated real-world sleep scenarios. Due to the limitations of the HSAT device, it is primarily suitable for identifying moderate to severe OSA patients (AHI ≥ 15), as recommended by AASM guidelines. In this study, the Soomirang device achieved a sensitivity of 100%, a specificity of 95.45%, and an accuracy of 97.14% for detecting moderate to severe OSA compared with ApneaLink Air^TM^. Furthermore, the AHI_Soomirang_ had a strong correlation and agreement with AHI_ApneaLink™_ (r = 0.95, average AHI difference = 3).

Comparisons with other devices highlight the competitive performance of the Soomirang device. Chen et al. validated a photoplethysmography (PPG)-based smartwatch for OSA screening, reporting an accuracy of 87.9%, a sensitivity of 89.7%, and a specificity of 86% when compared with HSAT for AHI ≥ 15 [22]. Similarly, Gu et al. demonstrated that the AHI derived from the Belun Ring strongly correlated with AHI from PSG (r = 0.894, average AHI difference = 23), with a sensitivity and specificity of 87.5% and 87%, respectively, for AHI ≥ 15 [35]. Papini et al. studied a smartwatch using a deep learning model trained on 250 recordings, achieving an AHI correlation of 0.61, a sensitivity of 59%, and a specificity of 90% for detecting moderate to severe OSA compared with PSG [21]. Yeh et al. reported that a smartwatch achieved an accuracy of 80.8%, a sensitivity of 93.1%, and a specificity of 73.5% for detecting moderate to severe OSA [14]. Additionally, a ring-based device utilizing an accelerometer and PPG signals to measure actigraphy, oxygen desaturation, pulse rate, and peripheral arterial tone showed a strong correlation with PSG (0.93–0.95) in a study of 167 participants [36]. Another patch-type device, which incorporated an electrocardiogram and a three-axis accelerometer to assess chest wall motion and calculate the chest effort index, achieved 80% sensitivity and 79.4% specificity for detecting moderate to severe OSA in a cohort of 119 patients [37]. Although direct comparisons across devices are challenging due to differences in the ground truth, our results indicate that the Soomirang device performs competitively or even better than many existing technologies. This improved performance may be attributed to several factors, including the incorporation of ST estimation in calculating AHI and the strong performance of the MLP-Mixer model.

Although the AHI is calculated as the ratio of detected apnea events to sleep time, this parameter is often underestimated or overlooked in wireless wearable devices for OSA detection, especially in systems that rely solely on deep learning algorithms. In this study, we estimated sleep time using accelerometer data as an additional input for AHI calculation. The sleep time estimated by the Soomirang device showed a strong correlation and good agreement with the evaluation time of ApneaLink Air™ (r = 0.9, average time difference = 7.5 min). This accurate estimation of sleep time played a crucial role in the high agreement of AHI values calculated by both devices. Gu et al. reported a ring-based platform that demonstrated a high correlation with PSG in total sleep time (r = 0.945, mean difference = 22 min) [35]. Fons Schipper et al. utilized chest-worn accelerometers to derive cardiac and respiratory signals for awake/sleep classification, achieving an accuracy of 93.3%, a sensitivity of 78.7%, and a specificity of 96.6% [11]. Incorporating additional bio-signals, such as respiration and pulse, into machine learning or deep learning models for epoch-to-epoch awake/sleep classification could further enhance the precision of sleep time estimation and, in turn, improve AHI calculations.

The MLP-Mixer model has been introduced and demonstrated good performance for apnea event detection. However, research using this model for OSA detection is still limited. This study supports the concept of the MLP-Mixer deep learning model for OSA detection. When compared with the widely used BiLSTM model, the MLP-Mixer showed a better AUC value for apnea event detection. This finding aligns with the results reported by Huang et al., namely that the multiscale MLP-Mixer outperformed the CNN and LSTM models for OSA detection in large datasets [25]. Due to its lighter weight, the MLP-Mixer could be a promising candidate for OSA detection, offering advantages over the commonly used CNN and BiLSTM models.

A wireless abdomen-worn sensor was utilized to monitor sleep status during sleep time. The device offers several advantages: its feasibility makes it highly convenient for home-based testing, its lightweight design minimizes sleep disturbance, and its repeatability and low cost make it suitable for large-scale OSA screening. However, the current study has notable limitations. First, the sample size was limited, which may affect the generalizability of the findings. While apnea event detection was conducted on an epoch basis to mitigate this limitation, the effectiveness of the Soomirang device for OSA detection on larger datasets needs further validation. Second, although ApneaLink Air™ served as a reliable reference for home-based sleep apnea detection, the gold standard for OSA diagnosis remains PSG, performed in a laboratory setting. To comprehensively validate the performance of the Soomirang device for OSA detection, in-lab studies using PSG as a benchmark are necessary.

## 6. Conclusions

This study presented an automated algorithm for detecting moderate to severe OSA using data collected from a wireless abdomen-worn sensor. The device integrates a capacitive sensor for measurement of abdominal movement and a three-axis accelerometer. By incorporating an adapted MLP-Mixer model and estimated ST, the proposed algorithm demonstrated excellent performance in detecting moderate to severe OSA when compared with an HSAT device. These results highlight the potential of this lightweight, wireless wearable device as a practical and effective tool for OSA screening. Future research should focus on recruiting larger datasets from both home-based sleep studies and sleep laboratory settings to further validate the effectiveness of the proposed algorithm and the performance of the Soomirang device in OSA detection.

## Figures and Tables

**Table 1:** Related works and their performance in sleep apnea detection and respiratory monitoring. PSG, polysomnography; RF, random forest; CNN, convolutional neutral networks; LSTM, long-short-term memory; BiLSTM, bidirectional long short-term memory; SA, sleep apnea; NP, nasal pressure; THO, thoracic respiratory effort; ABD, abdomen respiratory effort; PPG, photoplethysmography; AHI, apnea–hypopnea index; r, correlation coefficient; AUC, area under curve; Acc, accuracy; Sens, sensitivity; Spec, specificity; ST, total sleep time; IRB, institutional review board; MAE, mean absolute error; Ref, references; AF, oronasal airflow.

Ref	Device	Technique	Signals	Application	Ground truth	Performance
[12]	Polygraphy (wired)	REF, LSTM, CNN, MLP	NP, SpO2, THO, ABD	SA (full-night)	Expert	Acc/Spe: 0.8941/0.8967 (all features); 0.5126/0.6879 (ABD)
[20]	Chest wearable belt (wired)	RF	THO	SA (full-night)	Expert	AHI: r = 0.78
[21]	Wrist-worn PPG (wireless)	CNN	PPG	SA	Expert	AHI: r = 0.61; Sens/Spec: 0.59/0.9 (AHI ≥15)
[22]	Smart watch (wireless)	-	PPG + oximetry	SA	Expert/ ApneaLink^TM^ Air	Acc/Sens/Spec: 0.879/ 0.897/0.86 (ApneaLink, AHI≥15); 0.811/0.765/1 (PSG, AHI ≥5)
[14]	Ring (wireless)	-	Oximetry, PPG, accelerometer	SA/ST estimation	Expert	Acc/Sens/Spec: 0.808/ 0.931/0.735 (AHI ≥ 15) AHI: r = 0.888; ST: r = 0.967
[18]	Tracheal accelerometer (wireless)	CNN	Neck movements	SA	Expert	Acc/Sens/Spec: 0.81/ 0.87/ and 0.84 (AHI ≥ 15); AHI: r = 0.86
[15]	Face patches (wireless)	CNN	EEG, EOG, EMG	SA	Expert	Acc: 0.885
[23]	PSG	LSTM, BiLSTM	NP, ABD, AF	Apnea event detection	Expert	NP: AUC = 0.917; ABD: AUC = 0.865 AF: AUC = 0.851
[16]	Capacitive belt sensor	-	Capacitive changes	Respiration monitoring	MP150 (no IRB)	RR: r^2^= 0.99; PPI: r^2^= 0.89
[17]	Capacitive strain sensor	-	Capacitive changes	Respiration monitoring	Telescopic actuator (no IRB)	RR: MAE= 1
Our study	Single-point contact-based capacitive sensor and accelerometer	MLP-mixer	Capacitive changes, body movements	SA, apnea event detection	ApneaLink^TM^ Air	Acc/Sens/Spec: 0.9714/1/0.9545 (AHI ≥ 15); AHI: r = 0.95; ST: r = 0.9 Apnea event detection: AUC = 0.8428

## Data Availability

The data presented in this study are available from the corresponding author upon reasonable request.

## Abbreviations

OSA	Obstructive sleep apnea
HSAT	Home sleep apnea test
ST	Sleep time
AHI	Apnea hypopnea index
AASM	American Academy of Sleep Medicine
AI	Artificial intelligence
CNN	Convolutional neural network
LSTM	Long short-term memory networks
MLP	Multilayer perceptron
GELU	Gaussian Error Linear Unit
BCE	Binary cross-entropy
BiLSTM	Bidirectional long short-term memory networks
TPR	True positive rate
FPR	False positive rate
AUC	Area under the curve
LOA	Limit of agreement
sSD	Standard deviation
ET	Evaluation time
PSG	Polysomnography

## Appendix A

**Table A1:** Hyperparameter tuning for the MLP-Mixer model. AUC, area under curve; BCE, binary cross-entropy.

Fix Parameters	Varied Parameters	AUC
Window size = 100, hidden units = 128, batch size = 500, learning rate = 0.0001, iteration = 50,000, optimizer: Adam, loss function: BCE, folds = 5.	layer = 2	0.8155
	layer = 3	0.8428
Window size =100, layer = 3, batch size = 500, learning rate = 0.0001, iteration = 50,000, optimizer: Adam, loss function: BCE, folds = 5.	hidden units = 32	0.8387
	hidden units = 128	0.8428
